# Meeting Summary: State and Local Implementation Strategies for Increasing Access to Contraception During Zika Preparedness and Response — United States, September 2016

**DOI:** 10.15585/mmwr.mm6644a6

**Published:** 2017-11-10

**Authors:** Charlan D. Kroelinger, Lisa Romero, Eva Lathrop, Shanna Cox, Isabel Morgan, Meghan T. Frey, Lee Warner, Kathryn M. Curtis, Karen Pazol, Wanda D. Barfield, Dana Meaney-Delman, Denise J. Jamieson

**Affiliations:** ^1^Division of Reproductive Health, National Center for Chronic Disease Prevention and Health Promotion, CDC; ^2^Division of Global Health Protection, Center for Global Health, CDC; ^3^Association of Schools and Programs of Public Health, Washington, D.C.; ^4^Division of Congenital and Developmental Disorders, National Center on Birth Defects and Developmental Disabilities, CDC; ^5^Office of the Director, National Center for Emerging and Zoonotic Infectious Diseases, CDC.

Zika virus infection during pregnancy is a cause of microcephaly and other serious brain abnormalities (*1*). To support state and territory response to the threat of Zika, CDC’s Interim Zika Response Plan outlined activities for vector control; clinical management of exposed pregnant women and infants; targeted communication about Zika virus transmission among women and men of reproductive age; and primary prevention of Zika-related adverse pregnancy and birth outcomes by prevention of unintended pregnancies through increased access to contraception.* The most highly effective,^†^ reversible contraception includes intrauterine devices and implants, known as long-acting reversible contraception (LARC). On September 28, 2016, the Association of Maternal and Child Health Programs (AMCHP) and CDC facilitated a meeting in Atlanta, Georgia, of representatives from 15 states to identify state-led efforts to implement seven CDC-published strategies aimed at increasing access to contraception in the context of Zika virus (*2*). Qualitative data were collected from participating jurisdictions. The number of states reporting implementation of each strategy ranged from four to 11. Participants identified numerous challenges, particularly for strategies implemented less frequently. Examples of barriers were discussed and presented with corresponding approaches to address each barrier. Addressing these barriers could facilitate increased access to contraception, which might decrease the number of unintended pregnancies affected by Zika virus.

Twenty-six participants representing 15 states and 11 local health departments and clinics^§^ were selected to attend the Atlanta meeting, based on successful implementation of at least two of the seven strategies for increasing access to contraception (Table 1). AMCHP conducted a premeeting web-based assessment among attendees on the use of specific approaches for implementing the seven strategies. Responses to the assessment represented 12 states, including 12 state-level participants and six local-level participants and service providers.^¶^ During the in-person meeting, participants discussed the premeeting assessment responses and identified approaches to addressing barriers and maximizing facilitators for increasing access to contraception in three AMCHP-facilitated discussion sessions. Representatives from federal agencies and maternal and child health (MCH) organizations participated and provided technical and scientific expertise.** CDC analyzed qualitative data (*3*) to identify state implementation approaches (Table 2) that addressed barriers to and facilitators of success for the seven strategies.

**TABLE 1:** State and local jurisdictional-level strategies and approaches for increasing access to contraceptive methods — Association of Maternal and Child Health Programs and CDC-sponsored premeeting assessment topics, Atlanta, Georgia, September 2016

Strategy	Potential approaches to implement strategy
**1. Facilitate partnerships among private and public insurers, device manufacturers, and state agencies**	Establish direct payment program to absorb acquisition and stocking costs
	Develop pharmacy contracts to obtain a limited number of LARC devices
	Develop pharmacy contracts to return unused and unopened LARC devices
	Develop pharmacy contracts to bill insurers directly for LARC devices
**2. Reimburse providers for the full range of contraceptive services**	Implement a payment policy to reimburse for the costs of screening for pregnancy intention
	Implement a payment policy to reimburse for the costs of client-centered counseling
	Implement activities to reduce barriers to supplies by using prestocked kits for immediate postpartum LARC insertion
	Implement a payment policy to reimburse for the actual cost of LARC devices to provide the full range of contraceptive methods
	Develop a payment policy for device insertion, device removal, device replacement, device reinsertion, and client follow-up
	Implement a payment policy for the costs of immediate postpartum LARC supplies, procedure, and follow-up
**3. Remove logistic and administrative barriers for contraceptive services and supplies**	Ensure all FDA-approved contraceptive methods are covered by state policy
	Eliminate requirement for prior authorization for LARC prescriptions in state payment plan
	Eliminate requirement for multiple visits with a health care provider before LARC prescription in state payment plan
	Eliminate step therapy requirements before LARC prescription in state payment plan
**4. Train health care providers on current insertion and removal techniques for LARC using evidence-based guidance**	Incorporate federal evidence-based contraceptive guidance into state family planning guidelines
	Collect data on adopted or continued use of most or moderately effective FDA-approved methods of contraception among women aged 15–44 years*
	Collect data on adopted or continued use of LARC among women aged 15–44 years
	Provide resources to train and inform health care providers on LARC insertion and removal techniques
	Provide resources to providers to dispel common misperceptions about LARC methods including: IUD and infertility; IUD and abortifacients; LARC and cancer; LARC and weight gain; LARC and adolescents; LARC and nulliparous women
**5. Support youth-friendly reproductive health services**	Train health care providers to provide youth with client-centered reproductive health services
	Provide teen-focused, culturally appropriate materials for clinic services
	Collaborate with clinics to encourage expanded availability of adolescent-friendly reproductive health services (e.g., weekend and/or extended hours, eliminating prerequisite screening)
	Promote protocols to protect against confidentiality breaches, specifically for adolescent patients (e.g., not disclosing Explanation of Benefits to parents of minors)
**6. Engage smaller or rural facilities including community health centers**	Provide funding to smaller or rural health care facilities and clinics to support increased access to contraceptive services
	Develop policies on contraceptive use for smaller or rural health care facilities
	Provide targeted resources on highly effective, reversible contraception for providers serving predominantly small or rural communities
**7. Assess client satisfaction with service provision and increase consumer awareness**	Provide resources to clinics to collect or analyze data that assesses women’s satisfaction with chosen contraceptive method(s)
	Engage in health promotion campaigns to increase consumer awareness about LARC methods

**Abbreviations:** Food and Drug Administration = FDA; intrauterine devices = IUD; long-acting reversible contraception = LARC.

* Moderately effective contraceptive methods include injectables, pills, patch, ring, and diaphragm. Approximately 6–12 pregnancies per 100 women using these methods will occur during the first year of typical use compared with the most effective birth control methods, which result in fewer than one pregnancy per 100 women during the first year of typical use.

**TABLE 2:** Barriers, facilitators, and approaches for implementing strategies to increase access to contraception — Association of Maternal and Child Health Programs and CDC-sponsored meeting, Atlanta, Georgia, September 2016

Strategy	Barriers	Facilitators	Potential approaches to addressing barriers and maximizing facilitators
**1. Facilitate partnerships among private and public insurers, device manufacturers, and state agencies**	Consistent personnel turnover across agencies; limited understanding of internal structure of other agencies and organizations	Centralized state health department structure that partners to disseminate devices and revised policies	Institutionalize partnerships among agencies and organizations regardless of structure and personnel changes; establish public-private partnerships with device manufacturers, payers, health centers
**2. Reimburse providers for full range of contraceptive services**	Bundled reimbursement rates and global fees for immediate postpartum LARC; policies prohibiting prescription for LARC and insertion during the same visit	Expanded definitions of provider groups for provision of comprehensive client-centered counseling	Enhance reimbursement for immediate postpartum LARC services (device insertion and device cost); train mid-level providers, paraprofessionals, and support personnel on contraceptive counseling
**3. Remove logistic and administrative barriers for contraceptive services and supplies**	Lack of knowledge on billing and coding for contraceptive services; preapprovals, multiple visits, and step therapy requirements for clients to receive LARC; additional barriers for populations including the undocumented, uninsured, or incarcerated women of reproductive age	Provider champions influence provision of contraceptive services at the state, health systems, facility, and clinic levels	Train billers and coders on procedures for reimbursement policies; develop payment mechanisms for populations with less access to services; develop policies for same-day LARC insertion; eliminate prior authorization, cost sharing, and other requirements to receive LARC potentially leveraging 340B pricing; engage provider champions
**4. Train health care providers on current insertion and removal techniques for long-acting reversible contraceptives**	Lack of providers to insert LARC including family physicians, pediatricians, nurses; lack of information on providers who insert LARC	Release of updated evidence-based clinical guidance; release of updated quality family planning services recommendations	Complete a needs assessment of family planning services throughout the state; train providers on newest insertion techniques
**5. Support youth-friendly reproductive health services**	Policies on Explanation of Benefits release to policy-holder; clinic hours during normal business hours; clinics located far from schools	Available teen-focused, culturally appropriate materials	Engage in youth-friendly feedback on services including youth advisory boards, mystery shoppers, social media; ensure confidentiality of adolescent contraceptive services by revising policies with payers and insurers; encourage client-centered contraceptive counseling and screening for pregnancy intention for adolescents
**6. Engage smaller or rural facilities including community health centers**	Remote clinic location impacts availability of contraceptives and providers	Increased availability of telemedicine/telehealth opportunities	Train personnel on billing and coding procedures for contraceptive methods; provide carve-out or subsidy funding for patient encounter, counseling, contraceptive device, and insertion
**7. Assess client satisfaction with service provision and increase consumer awareness**	Lack of data on client satisfaction with contraceptive method; limited funding for state-level social media or traditional media campaigns	Examples of successful social media or traditional media campaigns for replication among other states	Distinguish between client satisfaction and experience; develop surveillance data on satisfaction and experience; collaborate with nontraditional partners including supermarket chains, retail outlets, and airports, to provide messaging on contraception particularly during emergency response

**Abbreviation:** long-acting reversible contraception = LARC.

**Strategy 1. Facilitate partnerships among insurers, manufacturers, and state agencies**. Respondents from eight of 12 states indicated that their state health agencies partnered to implement a direct payment program to absorb contraceptive device acquisition and stocking costs, develop pharmacy contracts to obtain devices, bill insurers directly for devices, or offer an option to return unused or unopened devices (Table 1). Personnel turnover and limited understanding of the internal organization and structure of other agencies were identified as barriers to developing contracts with payers and manufacturers (Table 2). Leaders with successful external partnerships leveraged current health department structures to institutionalize interagency partnerships and implement public-private partnerships to address these barriers.

**Strategy 2. Reimburse providers for the full range of contraceptive services**. Seven of 12 states implemented policies to reimburse providers for the actual cost of LARC devices and eliminate barriers in state payment plans for LARC prescriptions (Table 1). States described implementing a payment policy for immediate postpartum LARC supplies, procedures, and follow-up, using prestocked kits. States discussed payment policies to reimburse providers for pregnancy intention screening and client-centered contraceptive counseling. Successful approaches to overcoming payment barriers included leveraging health department support in developing immediate postpartum LARC policies that reimburse for the device costs, insertion fees, and training of mid-level providers on contraceptive counseling (Table 2).

**Strategy 3. Remove logistic and administrative barriers for contraceptive services and supplies**. Nine of 12 states implemented approaches to eliminate requirements for prior authorization, multiple visits, and step therapy^††^ approaches (Table 1). State-reported successful implementation approaches included training of billers and coders on reimbursement procedures, leveraging existing billing mechanisms (e.g., 340B pricing^§§^), eliminating requirements for multiple visits to facilitate same-day insertion, removing barriers for vulnerable or targeted populations, and engaging provider champions^¶¶^ to influence provision of services (Table 2).

**Strategy 4. Train health care providers on current insertion and removal techniques for LARC, using evidence-based guidance**. Ten of 12 states described integrating evidence-based contraceptive guidance into state family planning guidelines, collecting data on the use of the most effective or moderately effective*** Food and Drug Administration (FDA)-approved contraceptive methods, and providing resources to dispel common provider misperceptions about LARC (Table 1). Successful approaches to initiating statewide training efforts included statewide service assessment and continuing training of providers on the newest evidence-based techniques (Table 2).

**Strategy 5. Support youth-friendly reproductive health services**. Most (11 of 12) states provided teen-focused, culturally appropriate materials, offered training to providers on the provision of youth-friendly services, encouraged expanded availability of youth-friendly reproductive health services, and ensured that confidentiality concerns of adolescents were addressed in state or clinic policies (Table 1). Meeting participants indicated that a successful implementation approach for this strategy included soliciting youth feedback on services through youth advisory boards, social media, and youth mystery shoppers^†††^ at service sites (Table 2). Additional approaches include ensuring adolescent confidentiality among payers and insurers and appropriate screening and counseling for this age group.

**Strategy 6. Engage smaller or rural facilities, including community health centers**. Nine of 12 states provided funding to smaller or rural health care facilities and clinics, and targeted resources for LARC at these facilities (Table 1). Approaches to addressing barriers included training staff on billing and coding procedures, and funding subsidies in small or rural clinics for the client encounter, counseling, device cost, and insertion fees (Table 2).

**Strategy 7. Assess client satisfaction with service provision and increase consumer awareness**. Four of 12 states provided resources to assess client satisfaction with the chosen contraceptive method or engaged in health promotion campaigns to increase consumer awareness about highly effective, reversible methods (Table 1). Implementation approaches included developing assessment measures that differentiated between client satisfaction and client experience, and collaborating with nontraditional partners (including supermarket chains, retail outlets, or airports) to expand the reach of contraceptive messaging (Table 2). Meeting participants highlighted limited funding for state-level social media or traditional media campaigns as the greatest barrier to implementation. Use of consumer awareness media campaigns with nontraditional partners was discussed for the dissemination of prevention messaging during a public health emergency, particularly in the context of Zika preparedness. For example, partnering of the state health department with international airports to include Zika virus travel-related guidance from CDC was noted as an effective strategy for Zika preparedness messaging that could also include information on contraception access.

## Discussion

Among the 12 state-level responses to the assessment, the majority indicated that their health departments are implementing strategy-specific approaches for facilitating partnerships among insurers and device manufacturers (eight states); removing logistic and administrative barriers for contraceptive services and supplies (nine); training health care providers (10); supporting youth-friendly services (11); and engaging smaller or rural facilities (nine). Fewer reported that their states are implementing strategy-specific approaches for reimbursing providers for the full range of contraceptive services (seven) or assessing client satisfaction and increasing consumer awareness (four).

In the context of Zika preparedness, reducing gaps in contraception access might help reduce the number of unintended pregnancies affected by Zika virus infection. States emphasized the importance of partnerships among state and federal health agencies, payers, device manufacturers, and clinics during an emergency response. Attendees also emphasized the importance of overcoming payment barriers to reimburse providers for the full range of services. Currently, many states bundle payment for contraception under one global fee, particularly for immediate postpartum LARC, limiting reimbursement for the full cost of a device and specific insertion procedures (*4*). State policies that allow reimbursement for comprehensive client-centered counseling services are always important, but particularly during an emergency response (*5*), as such policies are implemented in part to prevent coercion of clients to choose any method, including LARC, by supporting informed, autonomous client decisions based on women’s individual needs and preferences (*6*).^§§§^ During an emergency response, it is also especially important to have straightforward and replicable campaigns to increase access to contraception to prevent unintended pregnancy. These are needed to support consumer-focused understanding by presenting the full range of reversible contraceptive methods, while describing the low maintenance and acknowledging the potential for side effects with LARC,^¶¶¶^ to increase awareness of contraceptive method options among all women of reproductive age, including adolescents. CDC works to develop evidence-based, clinical guidance during emergencies. During facilitated discussion, states requested detailed CDC response plans for increasing access to contraception to prevent unintended pregnancy, a primary strategy to reduce Zika-related adverse pregnancy and birth outcomes.

Participants emphasized that provider champions can increase both provision of LARC and training of clinical fellows and residents in current insertion and removal techniques. As service providers in the health care system, provider champions are well positioned to educate Medicaid agencies about the benefits of immediate postpartum LARC (*7*), take the lead in disseminating these practices to smaller and rural facilities, and serve as potential trainers and mentors to other providers. Participants discussed developing statewide provider networks to disseminate information and identify service providers who require additional training, and targeting training initiatives using evidence-based contraceptive guidance (*8*–*10*). Similar assessments have identified “contraceptive deserts,” defined as U.S. counties with fewer than one clinic for every 1,000 women in need of publicly funded contraception, further highlighting the need for contraceptive service availability.****

The findings in this report are subject to at least three limitations. First, information on contraceptive access was obtained from a relatively small number of persons from invited states; analysis and subsequent approaches developed from information gathered might not be generalizable to all jurisdictions. Second, data collected from participants were self-reported, and therefore, do not necessarily represent official state policies or all activities occurring in a state. Finally, approaches to increasing contraception access developed by participating states have not been evaluated by other states to ensure applicability in all settings. Some approaches implemented by participating states might not be appropriate or successful in other jurisdictions.

For women who choose to delay or avoid pregnancy during the Zika virus outbreak, access to the full range of reversible contraceptive methods in all health care systems increases their options to prevent unintended pregnancies. States are encouraged to include strategies to increase access to contraceptive services as a primary strategy in Zika preparedness plans, to prevent adverse pregnancy and birth outcomes associated with Zika virus infection in pregnancy.

**Summary**

**What is already known about this topic?**

Zika virus infection during pregnancy is a cause of congenital microcephaly and other brain abnormalities. Preventing unintended pregnancy during the Zika virus outbreak is one primary strategy to reduce the number of pregnancies affected by Zika virus. Sexually active women of reproductive age and their sex partners who choose to delay or avoid pregnancy during the Zika virus outbreak should have access to all FDA-approved contraceptive methods, including highly effective, long-acting reversible contraception; however, barriers limit access and availability. CDC has outlined seven strategies states can implement to increase access to contraceptive services.

**What is added by this report?**

On September 28, 2016, a meeting of 26 representatives from 15 jurisdictions was convened in Atlanta, Georgia to identify state-led efforts to implement the seven strategies. The majority of participants’ states implemented strategies facilitating external partnerships, removing logistic and administrative barriers, training providers, supporting youth-friendly services, and engaging smaller or rural facilities. A smaller proportion implemented strategies for increasing provider reimbursement, assessing client satisfaction, and increasing consumer awareness.

**What are the implications for public health practice?**

State-led approaches for implementing the seven strategies provide examples that can inform and support adoption in other jurisdictions in the context of Zika preparedness. These approaches could further facilitate access to contraception, which might decrease the number of unintended pregnancies affected by Zika virus infection.

## Footnotes

* https://www.cdc.gov/zika/public-health-partners/cdc-zika-interim-response-plan.html.

† Highly effective contraceptive methods result in a low pregnancy rate (i.e., fewer than one in 100 women using these methods will become pregnant during the first year of typical use).

§ A total of 15 state-level participants represented Colorado, Delaware, Georgia, Florida, Iowa, Louisiana, South Carolina, Tennessee, Texas, and Washington. There was one representative for each state health department, with Colorado, Florida, Iowa, Louisiana, and Texas having two state-level representatives. A total of 11 local-level participants represented urban areas and/or clinics in Arizona, Colorado, Florida, Georgia, Nebraska, New Mexico, New York, Texas, Washington, and Wisconsin. There was one representative for each local jurisdiction, with Colorado having two local-level representatives.

¶ A total of 12 states were represented in responses, including Arizona, Colorado, Delaware, Florida, Iowa, Louisiana, Nebraska, New York, South Carolina, Texas, Washington, and Wisconsin. Each state had one response, except three responses were received from Florida, and two responses each were received from Colorado, Iowa, Texas, and Washington, for a total of 18 responses overall. All respondents were state-level personnel except those from Washington, which included personnel at the state and local levels. Georgia, New Mexico, and Tennessee did not provide responses to the premeeting assessment.

** Participating federal agencies include CDC, Centers for Medicaid and Children’s Health Insurance Plan Services/Centers for Medicare and Medicaid Services, Bureau of Primary Health Care/Health Resources and Services Administration, Food and Drug Administration, and Office of Population Affairs. Participating MCH organizations included AMCHP, American College of Obstetricians and Gynecologists, Association of State and Territorial Health Officials, CityMatCH, March of Dimes, National Family Planning and Reproductive Health Association, and National Association of City and County Health Officials.

†† Step therapy is a type of prior authorization requiring that prescriptions be filled with the most cost-effective drug therapy (i.e., generic) progressing to more costly or risky drug therapies as necessary. The generic drug options for long-acting reversible contraception (LARC) are limited.

§§ 340B pricing is a discount drug program that requires drug manufacturers to provide outpatient drugs to eligible health care organizations/covered entities at significantly reduced prices. https://www.hrsa.gov/opa/index.html.

¶¶ Provider champions are persons who dedicate themselves to overcoming resistance or indifference to a beneficial intervention by supporting, publicizing, and implementing strategies to bridge the gap between knowledge and clinical practice.

*** Moderately effective contraceptive methods include injectables, pills, patch, ring, and diaphragm. Approximately six to 12 per 100 women using these methods will become pregnant during the first year of typical use.

††† Youth mystery shoppers for contraceptive services are adolescents hired by an organization or agency (health department) to visit clinics posing as new patients to collect information on clinic flow, resources provided, and the quality of interaction with clinic personnel and providers.

§§§ https://www.fpntc.org/training-and-resources/zika-toolkit-for-healthcare-providers-version-3-providing-family-planning.

¶¶¶ https://thenationalcampaign.org/sites/default/files/resource-primary-download/whoops_proof_insights.pdf.

****
https://thenationalcampaign.org/deserts .
